# The Italian Osteopathic Practitioners Estimates and RAtes (OPERA) study: A cross sectional survey

**DOI:** 10.1371/journal.pone.0211353

**Published:** 2019-01-25

**Authors:** Francesco Cerritelli, Patrick L. S. van Dun, Jorge E. Esteves, Giacomo Consorti, Paola Sciomachen, Eleonora Lacorte, Nicola Vanacore, for the OPERA-IT Group

**Affiliations:** 1 Clinical Human-based Research Department, Foundation COME Collaboration, Pescara, Italy; 2 Belgium National Centre, Foundation COME Collaboration, Mechelen, Belgium; 3 Gulf National Centre, Foundation COME Collaboration, Riyadh, KSA; 4 Instituto Piaget, Lisbon, Portugal; 5 Registro degli Osteopati d’Italia, Milan, Italy; 6 National Institute of Health, Rome, Italy; University of Notre Dame Australia, AUSTRALIA

## Abstract

The prevalence of osteopathic practitioners, their professional profile and features of their clinical practice, particularly where statutory regulation does not yet exist, are still significantly underreported. The Osteopathic Practitioners Estimates and RAtes (OPERA) project was developed as an European-based census dedicated to profiling the osteopathic profession across Europe. The present study aimed to describe the osteopathic practitioners and the profession in Italy. A voluntary, online based, closed-ended survey was distributed across Italy in the period between February and June 2017. An e-based campaign was set up to reach the Italian osteopathic professionals. Participants were asked to complete the forms by filling in the information regarding the demographics, working status and professional activities, education, consultation fees, patient complaints, treatment and management. The survey was completed by 4816 individuals. 196 people started the survey but did not finish, which corresponds to a 4% attrition rate. The majority of respondents were males (66.7%). The modal age group was 30–39 (40.0%). 73.8% of respondents had a previous academic degree, mainly in the fields of sports science (36.4%) and physiotherapy (25.3%). 25.6% declared not to have a previous academic degree. The majority of respondents declared to work alone (58.4%), while the remaining declared to work in association with other professionals. The osteopaths /citizens ratio was 8.0 osteopaths/100,000 citizens. The profile of osteopaths in Italy seems to be characterised by a self-employed young adult male working mostly as a sole practitioner, who has been trained as osteopath through a part-time curriculum and had a previous degree mostly in the fields of sports science or physiotherapy. These results provide important insights into the osteopathic profession in Italy. The varied professional educational backgrounds need to be considered with regard to the implementation of a professional licensing process and future pre-registration education in the country. The number of respondents is an estimate of the actual number of Italian osteopaths. Only the completion of the regulatory process and the creation of the mandatory official register will allow to know the number of Italy based osteopaths.

## Introduction

Modern healthcare practice requires the development and implementation of systems and policies which effectively scrutinise practitioners and healthcare providers in order to deliver safe and optimal patient care [1, 2]. In order to further advance the osteopathic profession and quality and its effectiveness of patient care, the Osteopathic International Alliance (OIA) is focused on obtaining reliable data regarding its scope of practice [3]. In a first attempt to identify the number of osteopaths worldwide, the OIA [4] asked the various national voluntary registers, professional associations and regulatory bodies in osteopathy, to provide estimates of the numbers of practitioners working in their respective countries. Although this was an important preliminary stage in describing the scope and characteristics of osteopathic practice, this approach produced a significant reporting bias, particularly in those countries where the unregulated nature of osteopathy is still a major concern [5]. In several countries where osteopathy is statutorily regulated, workforce surveys are regularly conducted [6–17]; however, the prevalence of osteopathic practitioners, their professional profile and features of clinical practice, particularly where statutory regulation does not exist, are still significantly underreported.

The Benelux study was the first study to objectively profile osteopathic practitioners in countries without statutory regulation in osteopathy [18]. Building upon the Benelux Osteosurvey tool [18], the Osteopathic Practitioners Estimates and RAtes (OPERA) project was developed as an European-based census dedicated to profiling the osteopathic profession across Europe. Arguably, it fulfils the need of patients, healthcare providers, and governmental and nongovernmental institutions across Europe to access up-to-date and reliable information regarding the geo-distribution, prevalence, incidence and profile of osteopaths in Europe. The OPERA study has been initially conducted in Italy and is currently underway in Spain, Andorra, Belgium, Luxembourg and Portugal.

Osteopathy is currently regulated in ten European countries: Denmark, Finland, France, Iceland, Liechtenstein, Malta, Portugal, Switzerland, Turkey and the UK [19]. In Italy, osteopathy has been recently recognized as a healthcare profession (Law 3/2018—Senate of Republic of Italy [20]). The Italian Register of Osteopaths (ROI), a voluntary registering body, has since its inception in 1989 contributed to improvements in professional standards of practice, education and training. ROI currently oversees the quality and standards of osteopathic education and training in 27 full-time and 45 part-time programmes in Italy [21]. Whereas part-time education and training programmes have a duration of six years and are only available to individuals with a degree in the fields of sports science, medicine and physiotherapy; full-time programmes in osteopathy have a duration of five years and can be attended by any individual with a high school diploma. Graduates from both programmes of study obtain a Diploma of Osteopathy (DO) and in some cases where Italian osteopathic education institutions have a degree awarded by a non-Italian university, students graduate with a Bachelor’s or a Master’s degree in osteopathy. The expectation in Italy is that osteopathic education programmes fulfil the WHO Benchmarks for Training in Osteopathy with 4,200 hours of training including a minimum of 1,000 hours of supervised clinical education [22]. Although formally defined, the benchmarks for osteopathic education were only established in 2010 and it is therefore challenging to reliably assess the quality of training and education prior to the publication and implementation of the WHO document and more recently the European Committee for Standardization (CEN) Standards of Osteopathic Healthcare Provision [23].

Osteopathy is becoming very popular in Italy and a recent study conducted by Eumetra Monterosa reported that over 10 million of Italians seek osteopathic care, particularly for musculoskeletal problems (70% of the reported reasons of the consultation). Interestingly, the 90% of the 800 Italians interviewed declared to be satisfied or fully satisfied with care received from an osteopath [24]. Within this national dichotomy created by the satisfaction of the general population from one side and the non-regulation from the other side, the OPERA study has been set up to profile the osteopathic profession in Italy and its practitioners. To this end, the aim of the study was to profile osteopathic practice in Italy by surveying osteopaths across the country regarding socio-demographic information, their practice and patient characteristics, presenting symptoms and clinical problems, use of diagnostic and treatment modalities, treatment outcomes and associated adverse events. In the present study a description of osteopathic professionals population in Italy will be presented.

## Materials and methods

### Objective

The present cross-sectional survey aimed to describe some features of osteopathic professionals in Italy. The SUrvey Reporting GuidelinE (SURGE) [25] was used as report guideline for this paper.

### Population

A voluntary, online based, closed-ended survey was distributed across Italy in the period between February and June 2017. Eligible participants were required to fulfil the following inclusion criteria: aged over 18 years old, successful completion of osteopathic training programme and practising as an osteopath. Any type of training leading to a Diploma in Osteopathy (DO) or equivalent was considered acceptable for inclusion. Exclusion criteria were: participation in a training course on single technique and/or osteopathic approaches, which did not lead to the award of an osteopathic title. This means that non-osteopaths could attend courses in e.g. craniosacral technique and call themselves osteopaths. As the aim of OPERA-IT was to use an online survey, professionals with no access to the online platform were excluded. Individuals who could not understand and respond in Italian and individuals with physical or mental impairments that precluded participation in the online survey were also excluded. Informed consent was given by participation in the survey as clearly stated in the survey presentation online page that every participants declared to have read and understood. The study was specifically approved by the Institutional Review Board of the Foundation COME Collaboration (12/2016).

### Recruitment

A dedicated website was created for the purpose of this study. An e-based campaign was set up to reach the Italian osteopathic professionals. Therefore, a combined social-media and newsletter strategy was implemented. A newsletter was sent by the largest osteopathic national voluntary registering body (ROI) to all its current members. ROI included approximately 2500 members and so, recruitment of participants from this source was considered not representative of the national osteopathic population. For this reason, an additional e-campaign was established to reach all osteopathic education institutions, voluntary registering bodies, professional associations and osteopathic internet providers/specialised websites (i.e., tuttosteopatia.it) asking them to forward an e-flyer advertising the study to their members. Moreover, all osteopathic education institutions participating with the OPERA study were provided with a paper-based flyer to be displayed at their location. Furthermore, a manual based search on white-pages was conducted to identify other sources of information. To encourage involvement, the e-flyer was sent to all the different mailing lists on twelve occasions, at weekly intervals, in the five-month recruitment period and data collection. The research team offered continuous professional development (CPD) webinars to participants after completing the survey. This was set up on a dedicated online platform accessible only with credentials. On the website, lectures were recorded and uploaded. Participants were able to log in at any time during the study period and watch the webinars.

### Survey tool

The OPERA study used a validated questionnaire [18] (S1 File and S2 File), which was translated following the forward-backward process recommended by the WHO by two bilingual English-Italian translators with experience in health demographic research. The questionnaire is composed of 57 questions and five sections collecting data on socio-demographics, osteopathic education and training, working profile, organisation and management of clinical practice and patient profile. A survey was piloted on twenty Italian-speaking osteopaths. Face-to-face interviews were conducted with all twenty osteopaths and public involvement panel to ascertain the understanding of items and visual presentations, after we amended the survey to increase comprehensibility. For the purpose of this present paper just the first three sections of the survey will be reported on–those dealing with practitioner training, demographics and practice location.

**Fig 1:**
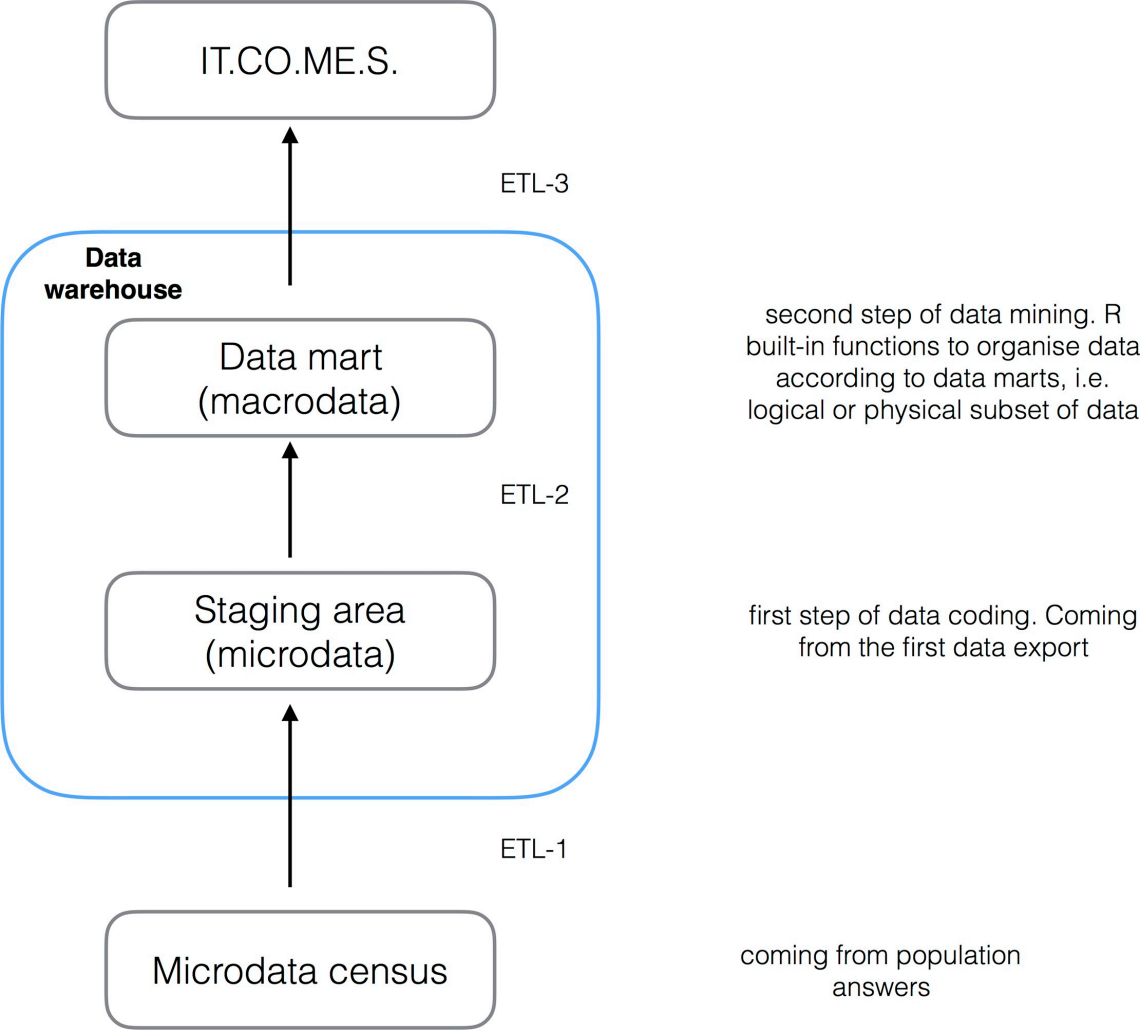
Data warehouse description.

The OPERA survey online platform, already developed and used, and an implemented data warehouse (IT.CO.ME.S) utilised for research purposes were used for this study (Fig 1). Data entered was encrypted with a symmetric keys procedure and sent over the internet using an ad-hoc software named COME Survey, which was developed specifically for this purpose to run highly secure surveys and studies containing potentially sensitive data. This system transfers data (microdata census) to a certified data centre (data warehouse–Fig 1); all information is processed and hosted in accordance with data protection regulations. Answers were anonymised and IP addresses were not disclosed to the research team. The system automatically managed the link between email address, StudyID and survey status, which means that research staff were not able to identify the responses provided and that double response was not allowed. Only OPERA research personnel had access to the complete, anonymised dataset.

### Privacy

OPERA uses a validated questionnaire carefully respecting the anonymity and privacy of data in accordance to the European directive 2002/58/CE of the European Parliament. Furthermore, all the information gathered was analysed and reported as grouped data. Completed questionnaires were individually examined and no attempt was made to identify respondents. Data will be stored for 5 years and used for further analyses and benchmarking.

### Information guidelines

Participants were asked to complete the forms by filling in the information regarding the demographics, working status and professional activities, education, consultation fees, patient complaints, treatment and management.

### Statistical analysis

The sample size was arbitrarily predicted and calculated summing all practitioners granted a Diploma in Osteopathy or equivalent from an Italian osteopathic education institution up to December 2016. This calculation also considered all the Italy based osteopaths who graduated in a foreign country and those who obtained an osteopathic diploma but not currently practising osteopathy. This produced an estimated sample of 5,100 osteopaths. Considering a standard deviation of 10%, it was predicted that the number of osteopaths in Italy ranged from 4,600 to 5,600. Taking into account that the survey response rate varied between 10 and 60% of those receiving the questionnaire [26] the number of practitioners who would participate at the survey was estimated between 460 to 3,300.

The analysis involving the Italian osteopathic practitioner population was based on the Italian National Institute for Statistics (ISTAT) data of January 2018 [27].

Data were analysed using different measures in relation to the type of data (continuous, ordinal, categorical and dichotomous). Mean, median, mode, point estimates, range, standard deviation and 95% confidence interval were used. For dichotomous measures, relative risk was used. Statistical analyses were based on a univariate and multivariate approach. R statistical programme (v. 3.1.3) was used to perform statistical analysis. A value of alpha less than 0.05 was considered as significant.

## Results

The survey was completed by 4816 individuals. 196 people started the survey but did not finish, which corresponds to a 4% attrition rate. 3,214 were male (66.7%), distributed 31.0% in North-west, 24.0% in the North-east, 25.0% in the Centre, 16.9% in the South, and 3.1% the Islands. The ratio was 8.0 DOs/100,000 citizens. A description of the distribution of DOs across the Italian macro-regions is shown in Table 1. The geographical distribution was based on the ISTAT distribution [26] and on the European nomenclature of territorial units for statistics (NUTS) [28] 71.0% of respondents declared to be part of at least one association or register. Almost all Italian osteopaths declared to be supportive of a healthcare regulation for the profession (95%).

**Table 1:** Geographical distribution.

	North-west	North-east	Centre	South	Islands	Tot
National distribution	1,493 (31.00)	1,156 (24.00)	1,204 (25.00)	813 (16.88)	150 (3.12)	4816
Rate osteopaths/ 100.000 citizens	9.28	9.93	9.99	5.80	2.25	7.96
Rate macroregion/national	1.16	1.25	1.25	0.73	0.28	1.00

Values represents N(%)

### Age and gender

**Fig 2:**
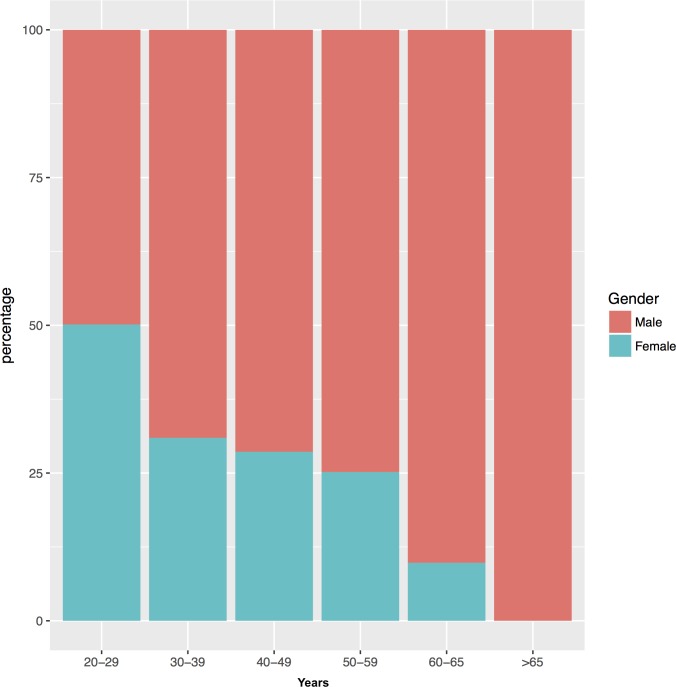
Bar chart of gender distribution across different age groups.

The majority of respondents were male (66.7%) (Table 2). Taking into consideration the age distribution, the highest represented age group was 30–39 (40.0%), whereas the over 65 group is represented by 0.3% of responders. Comparing the prevalence of the over 65s and the younger adults (20–29) as per the turnover index, it showed that for every over 65, there are 66 younger adults. Extensive data on gender per age distribution is shown in Fig 2.

**Table 2:** Demographics of the general osteopathic population.

		N	%
Gender	Female	1602	33.26
	Male	3214	66.74
Age Group	20–29	1045	21.7
	30–39	1928	40.03
	40–49	1119	23.24
	50–59	596	12.38
	60–65	112	2.33
	>65	16	0.33

Values represents N(%)

### Osteopathic training and lifelong learning

73.8% of respondents had a previous academic degree, mainly sports science (36.4%) and physiotherapy (25.3%). 25.6% declared not to have a previous academic degree. The most prevalent osteopathic education and training is 6 years part-time (66.6%) at an Italian osteopathic education institution (95.0%). The vast majority of respondents attend CPD courses (93.0%). On average the number of courses attended is 2 (range 1–12) over the past year. Moreover, approximately 2 out of 3 respondents allocate between 25–50% of their working time to continued professional development (e.g. osteopathic technique CPD) and scientific updates (e.g. autonomous reading of scientific papers). Table 3 reports the descriptors on professional training.

**Table 3:** Descriptors on professional training.

		N	%
Type of training	Part-time	3,207	66.59
	Full-time	1,609	33.41
Continuous Professional Development	No	341	7.08
	Yes	4,475	92.92
Percentage of working time dedicated to scientific updating	0	99	2.06
	1 to 5	1,063	22.07
	6 to10	1,528	31.73
	11 to 25	1,341	27.84
	26 to 50	627	13.02
	51 to 75	158	3.28
Percentage of working time dedicated to professional updating	0	17	0.35
	1 to 5	275	5.71
	6 to10	819	17.01
	11 to 25	1,352	28.07
	26 to 50	1,463	30.38
	51 to 75	890	18.48
Previous qualification	Physiotherapy	1,218	25.29
	Massage therapy	583	12.11
	Sports science	1,755	36.44
	Other	583	12.11
	None	1,231	25.56
Type of osteopathic degree	Other osteopathic degree	2,258	46.89
	D.O.	4,424	91.86
	D.O. (USA)	37	0.77
	Eur Ost D.O.	65	1.35
	R.O.	5	0.1
	B.Ost.	71	1.47
	M.Ost.	11	0.23
	BSc	617	12.81
	MSc	249	5.17
	Doctorate	349	7.25
	Ph.D.	49	1.02
	other	95	1.97

Values represents N(%)

### How osteopaths work

The majority of respondents declared to work as sole practitioners (58.4%), while the remaining declared to work in association with other professionals such as other osteopaths, general practitioners, physiotherapists, psychologists, speech therapists, dieticians, dentists, massage therapists, physicians with specialty, optometrists, others.

Taking into consideration the distribution among macro-regions, the trend to work single handed is lower in the center (51.3%) compared to the other macro-regions: North-west (59.1%), North-east (61.8%), South (61.9%), Islands (64.0%).

## Discussion

This study profiled osteopathic professionals in Italy by surveying osteopaths across Italy regarding their practice and demographic information. The Osteopathic Practitioners Estimates and Rates (OPERA-IT) was successfully as the first national survey relevant to osteopathy in Italy. Analysis of data provided by the respondents who completed the questionnaire yields important new findings relating to osteopathic practitioners and inter-professional collaborations that may not be readily observed through other national health care data sets. The results of this study provide a description of the osteopathic population categorised in terms of gender and age therefore providing a general profile of osteopathic practitioners and the profession in Italy. In general, the typical osteopath in Italy is male aged between 30–39 years old, living in the North of Italy with a previous academic degree, mainly in physiotherapy and sports science, and a 6-year part-time osteopathic education and training, working as self-employed osteopath in a private practice. The results show a gender shift towards female practitioners of the osteopathic profession in Italy during the last years (Fig 2). This trend is in line with the gender shift towards female practitioners of health professions in general [29]. The number of respondents is an estimate of the actual number of Italian osteopaths. According to our estimate, the number of respondents may be considered a representative sample to describe the population of Italy based osteopaths. Only the completion of the regulatory process and the creation of the mandatory official register will allow to know the number of Italy based osteopaths. Only the completion of the regulatory process and the creation of the mandatory official register will allow to know the number of Italy based osteopaths.

Compared to other surveys, this survey showed comparable characteristics of osteopathic practitioners. Indeed, the Benelux study demonstrated a similar prevalence of gender (male) within 30–39 years old [18], whereas the latest 2011 KPMG Report [10] showed that osteopaths in UK are older. However, this is only true if we consider the entire Benelux, whereas the Belgian osteopathic demography is very similar to the UK one. The majority of the respondents in Belgium and UK are aged between 30–49 (56.4% and 58.9% respectively) whereas the 31.8% of the UK osteopaths are older than 50. From an educational standpoint, the results of this current study are similar to those from the Benelux study, where the majority of osteopaths received part-time osteopathic education (80%) and had mainly a previous physiotherapy degree (85%). However, the percentage of Italian osteopaths with a previous physiotherapy degree is very different from the Benelux ones (25% vs 85%). Taking into account full-time osteopathic education, one third (33.4%) of Italian osteopaths declared to have undertaken a 5-year full-time osteopathy programme. This finding seems to be higher compared to the general Benelux result (20%), doubled compared to the Benelux subgroups of the Belgian French community (9%) and Dutch community (12%) but almost identical compared to the Flanders community (34%). However, when previous academic title is taken into consideration, a quarter of Italian respondents declared not to have a previous academic degree. This result is in contrast with the Benelux study where only the 9% of the participants declared not to have an academic title. As a potential consequence, a critical assessment of these results might open further discussion regarding the title and the type of diploma (training) in osteopathy, particularly relevant in those European countries where this professional title has not been recognised yet by the ministry of health/education as an academic qualification.

Another relevant element of the profile of Italian osteopaths is the multidisciplinary and interdisciplinary collaboration. Almost half of the sample (41.6%) claimed that they are associated to other professionals, thus suggesting an inter-professional collaboration network. Compared to a similar study conducted among Swiss osteopaths [30], Italy based osteopaths demonstrate a slightly higher inter-professional collaboration ratio (41.6% vs 39.9%).

As a general attitude, almost all Italian osteopaths seem to be supportive of a healthcare regulation for the profession (95%), that, in accordance to their expectations, might positively influence professional collaboration with other healthcare professionals and potentially quality of care of their patients.

The impact of this first OPERA-IT survey can be predicted in several aspects. Firstly, having a defined profile of osteopathic professionals might create opportunities for critical and informed discussions with policy makers and other stakeholders involved in the regulatory process for the profession in Italy. Current data can be utilised to tailor the regulatory strategies based on policy outcomes (taking into account those professionals who did not have a former academic background). Second, there are benefits for professional associations and registers: in order to create adequate within-professional improvement regulatory strategies or to be up-to-date on the prevalence of the profession nationally. Third, there are advantages for osteopathic practitioners: to be informed, for example, regarding the geo-distribution of osteopaths. Fourth, taking into account other healthcare professionals, this survey highlights the positive attitude in osteopathy towards multidisciplinary clinical collaborations. Finally, from a patient standpoint: a better and more precise vision of the Italian osteopathic practitioner profile.

### Strengths and weaknesses of this study

To the best of our knowledge, no other study has specifically investigated the osteopathic profile and characteristics in Italy. Indeed, this study is the first in Italy to estimate the prevalence of osteopathic practitioners in the Italian territory using large nationally representative data, to identify the profile of Italian osteopaths.

However, this study did not allow us to survey all the osteopaths working in Italy. This implies that the data presented might be underestimated. Second, the survey was subject to bias from both its geographical and contextual deployment, and as a self-report could have been influenced by responder bias. Third, since the invitation to participate to the survey was not personally addressed it was impossible to calculate the response rate. Finally, our data is specific to Italy and therefore cannot be generalized for the osteopathic profession in other countries.

## Conclusions

The profile of osteopaths in Italy seems to be characterised by a self-employed young adult male working mostly single-handed, who has had a part-time osteopathic education and a previous degree mostly in the field of sport science or physiotherapy. The results of this study provide important insights into the osteopathic profession in Italy. The varied professional educational background needs to be taken into consideration with regard to the implementation of a professional licensing process and future pre-registration education in the country. Further follow-up studies have been planned to track future changes within the osteopathic profession.

## Supporting information

S1 File OPERA questionnaire English version. (PDF)

S2 File OPERA questionnaire Italian version. (DOCX)
