# Supplementary material for: The Italian Osteopathic Practitioners Estimates and RAtes (OPERA) study: A cross sectional survey
Source: PLoS One. 2019 Jan 25;14(1):e0211353. doi: 10.1371/journal.pone.0211353 (PMC6347434; doi:10.1371/journal.pone.0211353)
Supplement: S1 File — (PDF) [file pone.0211353.s001.pdf]

OPERA Questionnaire

(English version)

General information

1) Gender

   a) female
   b) male

2) Age

   a) 20-29
   b) 30-39
   c) 40-49
   d) 50-59
   e) 60-65
   f) >65

3) In case of pathology, please choose the appropriate. More than one answer can be ticked
   a) Physical disability
   b) Sensory disability
   c) Chronic disease
   d) Genetic or congenital disease

4) Nationality

   a) List

5) Are you a member of a professional association for osteopathy?

   a) yes
   b) no

   5 A) If so, which one
   (Which professional association are you a member of?)

   a) List
   b) Other : …

5) Are you included in a register?

a) yes
b) no

5 A) If so, Which register are you included in? more than one answer allowed

a) List of countries

Education

6) Where did you have your osteopathic training? More than one answer can be ticked.

a) Italy
b) Abroad

7) Which year did you receive your degree/diploma of osteopathy?

a) 1970
b) ...
c) …
d) 2016

8) If in Italy, in which school? More than one answer can be ticked.

a) List of Italian OEI's
b) Other

9) If abroad, in which country?

a) France,
b) UK,
c) Spain,
d) Germany,
e) Switzerland,
f) Austria,
g) Croatia,
h) Belgium,
i) The Netherlands,
j) Finland
k) Norway
l) Sweden
m) Denmark
n) USA
o) Australia
p) New Zealand
q) Portugal

r) Russia
s) Poland
t) Brasil
u) Argentina

10) Did you receive a full-time or a part-time osteopathy education?

a) Full-time education
b) Part-time education

10A: How long was the programme (in years)?
1. 1
2. 2
3. 3
4. 4
5. 5
6. 6

11) Did you complete the clinical exam and your dissertation/thesis in the same year? ?
a) Y
b) N

11 A) if N: in which year did you complete both? list of years

12) Which preliminary training did you have prior to your osteopathy education? More than one answer can be ticked.

a) physiotherapy
b) medicine',
c) massage',
d) sport science
e) nursing',
f) midwifery',
g) veterinary',
h) psychology',
i) biology',
j) occupational therapy
k) dentistry
l) other health care
m) other non-health care
n) none',

13) Which titles/names do you bear related to osteopathy? More than one answer can be ticked.

a) Osteopath

b) D.O.
c) D.O. (USA)
d) Eur Ost D.O.
e) M.R.O.
f) C.O.
g) B.Ost
h) M.Ost
i) BSc
j) MSc,
k) Dr.
l) Ph.D.
m) other

13 A) 'Please specify other'

14) In terms of professional improvement, do you attend CPD courses?

a) yes
b) no
    i) If yes; How many hours during the year (on average)?

15) In terms of professional improvement, What is the percentage of your time dedicated e.g. discussion with colleagues and/or with other healthcare professionals, reading scientific papers, personal study)??

a) 0%
b) <5%
c) <10%
d) <25%
e) <50%
f) <75%
g) >75%

Practice organisation and fee

15) How many years have you been working as an osteopath?

16) In addition to osteopathy, do you carry out other professional activities?

a) yes
b) no

16 A) Which professional activities? More than one answer can be ticked.

- physician
- physiotherapist

- massage
- sport science',
- nurse',
- sport coach,
- midwife',
- veterinary',
- biologist',
- 'psychologist',
- occupational therapist
- 'osteopathy school administrator',
- 'general director/department of an osteopathic school',
- 'lecturer in an osteopathy education school',
- 'tutor in an osteopathy education school',
- 'supervisor trainees/dissertations',
- 'post-graduate lecturer within the framework of continuous education'
- 'occasionally lecturer in one or more osteopathic school',
- 'Administrator of company',
- Board member of professional association/register
- 'other',
  - o 16 B) 'specify other',

17) How many days per week do you work with patients, as an osteopath?

18) How many hours per week do you work with patients, as an osteopath?

19) Do you work as (more than one answer can be ticked):

a) Employee in paid employment
b) Independent
c) Independent, not in own practice

20) Where do you work? More than one answer can be ticked.

a) Private practice
b) Hospital/health care centre
c) Osteopathic education institute
d) University or College
e) Other :
   i) 20 A) Specify other

21) Do you work alone or in a group practice for osteopathy? More than one answer can be ticked.

a) Alone
b) Group practice

22) How many osteopaths work in this osteopathic group practice?
a) 2
b) 3

c) 4
d) 5
e) >5

23) If you work in a multidisciplinary centre, which other professions are involved? More than one answer can be ticked.
a) osteopath',
b) general practitioner/family doctor,
c) physiotherapist',
d) occupational therapist
e) psychologist',
f) speech therapist',
g) Dietician/nutritionist',
h) Dentistry',
i) Massage',
j) medical specialists ',
k) other',
    i) 23 A) specify other

24) In which Spanish province do you work? More than one answer can be ticked.
a) List of Spanish provinces

25) Do you work in different countries? Yes/No

25 A) If Y, In which other country do you also work as an osteopath?
b) List of countries

25) How much time do you plan for a new patient?
a) < 30 min.
b) 30 min. - 45 min.
c) 46 min. - 60 min.
d) > 60 min.

26) How much time do you plan for a returning patient?
a) < 30 min.
b) 30 min. - 45 min.
c) 46 min – 60 min.
d) > 60 min.

27) Do you conduct a new diagnostic examination with each consultation?
a) Yes
b) No
c) Not always

28) Do you make use of exclusion diagnostics to determine whether or not you will treat a patient?
a) Yes
b) No

29)Which diagnostic techniques do you use? If a technique is unknown, tick "never".

0 : never 1: seldom 2: regularly 3: often 4: always

**Diagnostic techniques**	**0**	**1**	**2**	**3**	**4**
assessment of visceral mobility	o	o	o	o	o
assessment of the cranium (neuro- and viscerocranium)	o	o	o	o	o
classic orthopedic tests	o	o	o	o	o
classic neurologic tests	o	o	o	o	o
fascial testing	o	o	o	o	o
inspection	o	o	o	o	o
muscle function testing	o	o	o	o	o
neurolymphatic reflex tests	o	o	o	o	o
palpation of position/structures	o	o	o	o	o
palpation of movement	o	o	o	o	o
percussion and auscultation	o	o	o	o	o
Range Of Motion (ROM)	o	o	o	o	o
tender points (Jones technique) and trigger points	o	o	o	o	o
otoscopy	o	o	o	o	o
"Combur test" (Urin analysis)	o	o	o	o	o

33 A) Which other non-listed diagnostic techniques do you use?

34)Which treatment techniques do you use? If a technique is unknown, tick "never".

0 : never 1: seldom 2: regularly 3: often 4: always

**Treatment techniques**	**0**	**1**	**2**	**3**	**4**
automatic shifting and fluid body approach	o	o	o	o	o
fascial techniques (e.g. myofascial release-, Chila-techniques, “unwinding”-techniques)	o	o	o	o	o
fluid techniques (e.g. lymphatic pump techniques)	o	o	o	o	o
functional techniques (e.g. Sutherland, Hoover, Jones or counterstrain techniques, Balanced Ligamentous Tension techniques (BLT))	o	o	o	o	o
general osteopathic articulations/ mobilisations (e.g. General Osteopathic Treatment (GOT), Total Body Adjustment (TBA))	o	o	o	o	o
“High Velocity Low Amplitude” (HVLA-techniques, impulse manipulations)	o	o	o	o	o
“Muscle Energy Techniques” (MET) (techniques with which neuromuscular	o	o	o	o	o

reflexes are used).					
neurocranial and viscerocranial techniques	o	o	o	o	o
neurovisceral and neurolymphatic reflex techniques	o	o	o	o	o
percussion and vibration techniques	o	o	o	o	o
Progressive Inhibition of Neuromuscular Structures (PINS)	o	o	o	o	o
soft and connective tissue techniques (muscular and connective tissue stretching, -friction, ...)	o	o	o	o	o
toggle-techniques	o	o	o	o	o
visceral manipulations	o	o	o	o	o
trigger points	o	o	o	o	o

34 A) Which other non-listed techniques do you use?

35) Do you make use of any of the following specific examination/treatment technique?

0 : never 1: seldom 2: regularly 3: often 4: always

**Intimate region techniques (diagnostics and/or treatment)**	**0**	**1**	**2**	**3**	**4**
Intra-oral	o	o	o	o	o
Breast	o	o	o	o	o
Vaginal	o	o	o	o	o
Rectal	o	o	o	o	o

36) Do you give advice to your patients? More than one answer can be ticked.

0 : never 1: seldom 2: regularly 3: often 4: always

	**0**	**1**	**2**	**3**	**4**
Exercises	o	o	o	o	o
ADL-advice (Activities of the everyday life)	o	o	o	o	o
Dietary/Nutritional advice	o	o	o	o	o
Ergonomic advice	o	o	o	o	o
Pain education	o	o	o	o	o

36 A) Which other non-listed advice do you use?

37) Are there patient groups with whom you prefer working in the practice?

a) Yes

b) No

37 A) With which patient groups do you prefer working?

- infants
- children
- pregnant women
- athletes
- elderly
- other :
  - o 37 B) specify

38) Do you use supplementary diagnostic and/or therapeutic methods, for which you were trained, in your osteopathic practice?

a) Yes
b) No

38 A) If so, please choose from the list below if the supplementary method was a diagnostic and/or therapeutic method? (at the right from this list a table where they have to mark D(iagnostical) and/or T(herapeutical))

A. Applied Kinesiology
B. Dry needling
C. Electrotherapy
D. Exercise therapy
E. Homeopathy
F. Manual therapy methods (e.g. Cyriax, Kaltenborn-Evjenth, Maitland, McKenzie, Mulligan, Van Der Bijl)
G. Medical imaging (e.g. ultrasonography, RX, …)
H. Nutrition therapy
I. Orthotics
J. Pharmacotherapy
K. Phytotherapy
L. Pilates
M. Psychotherapy
N. Steroid/analgesic injections
O. Taping
P. Traditional Chinese Medicine (e.g. acupuncture)

39) How do you give prominence to your practice? More than one answer can be ticked.

a) Personal internet site
b) Leaflets
c) Collaboration with other health care providers
d) Advertisements

e) Visiting cards
f) Mention on the list of the professional register
g) Publications in magazines, newspapers, etc.
h) social media (e.g. twitter, facebook)
i) conference
j) Other :
    i) 39 A) Please, specify other

40) Do you charge different fees for different patient populations?

a) Yes
b) No

41) How much do you charge for a first consultation (VAT excluded)? If you charge different fees, please indicate the highest fee.
a) <25 euro,
b) 26-30',
c) 31-39',
d) 41-50',
e) 51-60',
f) 61-70',
g) 71-80',
h) 81-90',
i) 91-100'
j) >100',

42) How much do you charge for the following consultation (VAT excluded)?
a) <25 euro,
b) 26-30',
c) 31-40',
d) 41-50',
e) 51-60',
f) 61-70',
g) 71-80',
h) 81-90',
i) 91-100'
j) >100',

43) Do you give a reduction in the event of payment problems?

a) Yes
b) No

Your patients

44) What is the average waiting period for a first appointment for a patient?

a) Same day
b) Within 1 week
c) 1-2 weeks
d) 2-3 weeks
e) 3-4 weeks
f) > 4 weeks

45) How many patients do you treat on average on a working day?
a) 0-5,
b) 6-10
c) 11-15
d) 16-20
e) >20,

46) How many patients do you treat on average per working week?
a) <5,
b) 6-10,
c) 11-15,
d) 16-20,
e) 21-25,
f) 26-30,
g) 31-35,
h) 36-40,
i) 41-45,
j) 46-50,
k) 51-55,
l) 56-60
m) 61-65
n) >65,

47) How many new registrations do you record on average per week? A new registration regards a patient who is not being treated at the moment, but who could already be a patient of yours.
a) 0-5,
b) 6-10
c) 11-15
d) 16-20
e) >20,

48) What is the average number of consultations per patient per year? Chronic patients not included.

49) Your patients are mainly:

a) male
b) female
c) equally divided
d) I do not know

50) Do you treat animals?

a) Y
b) N

50 A) if Y: How many hours (on average) per week?

51)How do the patients come to you? More than one answer can be ticked.

0 : never 1: seldom 2: regularly 3: often 4: always

	**0**	**1**	**2**	**3**	**4**
Referral by a physician	o	o	o	o	o
Referral by a physiotherapist	o	o	o	o	o
Referral by a colleague-osteopath	o	o	o	o	o
Referral by another professional	o	o	o	o	o
On advice of another patient	o	o	o	o	o
On advice of an acquaintance	o	o	o	o	o
On the patient's own initiative	o	o	o	o	o

51 A) What are the other ways by which your patients come to you?

52) In the past 6 months you treated patients from the age group:
More than one answer can be ticked.

a) Younger than 6 months
b) 6 months to 2 years
c) 2 to 10 years
d) 11 to 20 years
e) 21 to 40 years
f) 41 to 64 years
g) 65 years and older

53) The majority of the patients is:
3 answers at the most.

a) Younger than 6 months
b) 6 months to 2 years
c) 2 to 10 years
d) 11 to 20 years
e) 21 to 40 years
f) 41 to 64 years
g) 65 years and older

54) For which complaints (per body region) do the patients consult you the most?

0 : never 1: seldom 2: regularly 3: often 4: always

	0	1	2	3	4
head	o	o	o	o	o
cervical spine	o	o	o	o	o
shoulder girdle	o	o	o	o	o
elbow	o	o	o	o	o
wrist/hand	o	o	o	o	o
thoracic spine	o	o	o	o	o
thorax	o	o	o	o	o
abdomen	o	o	o	o	o
lumbar spine	o	o	o	o	o
pelvis	o	o	o	o	o
hip	o	o	o	o	o
knee	o	o	o	o	o
ankle/foot	o	o	o	o	o

55) For which more specific complaints did the patients consult you the most in the past year?

0 : never 1: seldom 2: regularly 3: often 4: always

	0	1	2	3	4
ankle sprain	o	o	o	o	o
cervicobrachialgia	o	o	o	o	o
complaints during / after pregnancy / childbirth					
craniomandibular complaints	o	o	o	o	o
baby colic	o	o	o	o	o
dermatological complaints (e.g. eczema, psoriasis, ...)	o	o	o	o	o
digestive disorders	o	o	o	o	o
dizziness	o	o	o	o	o
earache	o	o	o	o	o
fibromyalgia	o	o	o	o	o
frozen shoulder	o	o	o	o	o
gastro-oesophageal reflux	o	o	o	o	o
headache and migraine	o	o	o	o	o
incontinence	o	o	o	o	o
irritable bowel syndrome	o	o	o	o	o
low back pain	o	o	o	o	o
meniscus injury	o	o	o	o	o
menstrual complaints	o	o	o	o	o
neck complaints	o	o	o	o	o
post-operative complaints	o	o	o	o	o
psychological complaints	o	o	o	o	o
sciatica	o	o	o	o	o
sleeping problems	o	o	o	o	o
stress related complaints	o	o	o	o	o
tiredness	o	o	o	o	o

55 A) What are the other more specific complaints, not listed, have you been contacted?

56) Please indicate how frequently you performed or encountered the following over the last month in your practice?

	never	rarely	occasionally	sometimes	frequently	usually	always
Monitoring the patient`s reaction to the treatment.	O	o	O	o	o	o	O
Mild adverse events during the consultation	O	O	O	O	O	O	O
Major adverse events during the consultation	O	o	O	o	o	o	o
Mild adverse events after the consultation	O	o	O	o	o	o	o
Major adverse events after the consultation	O	O	O	O	O	O	O
Referral to GP	O	O	O	O	O	O	O
Referral to other healthcare professionalsvg fg	O	O	O	O	O	O	O
Referral to medical specialists	O	O	O	O	O	O	O

How much do you agree with each statement about your view about osteopathy in Italy?

	strongly disagree	disagree	somewhat disagree	neither agree or disagree	somewhat agree	agree	strongly agree
Medical professionals (Medical doctors, hospital	o	o	o	o	o	o	o

consultants etc.) I come into contact with take osteopathy seriously.							
Overall, I believe the quality of patient care provided by osteopaths in Italy is good	o	o	o	o	o	o	o
Osteopathy should be regulated by law.	o	o	o	o	o	o	o
Regulation would have a positive effect on how I practice as an osteopath.	o	o	o	o	o	o	o
Patients should be better reimbursed for osteopathic care	o	o	o	o	o	o	o
I would like to have a better cooperation with other healthcare professionals	o	o	o	o	o	o	o
	o	o	o	o	o	o	o
	o	o	o	o	o	o	o
