# Supplementary material for: The Italian Osteopathic Practitioners Estimates and RAtes (OPERA) study: A cross sectional survey
Source: PLoS One. 2019 Jan 25;14(1):e0211353. doi: 10.1371/journal.pone.0211353 (PMC6347434; doi:10.1371/journal.pone.0211353)
Supplement: S2 File — (DOCX) [file pone.0211353.s002.docx]

OPERA

**Informazioni generali**

1. Sesso
   1. Donna
   2. Uomo
2. Anni
   **a**. 20-29
   **b**. 30-39
   **c**. 40-49
   **d**. 50-59
   **e.** 60-65
   **f.** >65
3. Nazionalità
4. Italiana
5. Europea
6. extra-europea
7. È membro di un’associazione nazionale di osteopatia o di un registro?
8. Si
9. no
   - 1. Se si quale? Consentita più di una risposta
10. ROI - registro degli osteopati d’Italia
11. FESIOS - Federazione Sindacale Italiana Osteopati
12. Associazione Professionale degli Osteopati
13. AMOI - Associazione Medici Osteopati Italiani
14. UPOI - Unione Professionale Osteopati d’Italia
15. AIOC - Associazione Italiana di Osteopatia Classica
16. BFD Italia - Associazione Osteopati di Bio-Meccanica Funzionale Dinamica
17. altro
18. È iscritto ad un registro estero?
19. Si
20. no
    - 1. Se si, quale? Consentita più di una risposta

**- Europa**:

1. Francia
2. Regno Unito
3. Spagna
4. Germania
5. Svizzera
6. Austria
7. Croazia
8. Belgio
9. Olanda
10. Paesi Scandinavi

- **Stati Uniti d’America**
- **Australia**

Formazione

1. Dove si è formato da osteopata?
2. Italia
3. Estero
4. In che anno ha conseguito il diploma di Osteopatia?
5. 1970
6. 1971
7. 1972
8. 1973
9. …
10. …
11. …
12. 2015
13. Se in Italia, in quale scuola? Consentita più di una risposta
14. I.I.O./I.S.O Istituto Italiano di Osteopatia/Istituto Superiore di Osteopatia
15. C.E.R.D.O. - Centre pour l’Étude, la Recherche et la Diffusion Ostéopathiques
16. A.I.O.T. - Accademia Italiana Osteopatia Tradizionale
17. E.I.O.M. - European Institute for Osteopathic Medicine
18. C.I.O. - Collegio Italiano di Osteopatia
19. SIOTEMA- Scuola Italiana di Osteopatia e Terapie Manuali
20. C.R.O.M.O.N. - Centro Ricerche Olistiche per la Medicina Osteopatica e Naturale
21. C.S.d.O.I. - Centro Studi di Osteopatia Italiano
22. E.O.P. - European Osteopathic Project
23. SOMA - Istituto Osteopatia Milano
24. A.T.S.A.I. - A.T. Still Academy Italia
25. C.R.E.S.O. - Centro Ricerche e Studi Osteopatici
26. Chinesis I.F.O.P. - Istituto di Formazione in Osteopatia e Posturologia
27. I.C.O.M.M. - International College of Osteopathic Manual Medicine
28. AbeOS - Abe Osteopathy School
29. TCIO - Scuola Osteopatia Milano
30. AISeRCO - Accademia Italiana per lo Studio e la Ricerca Clinica in Osteopatia
31. Osteopathic College
32. CSOT - Centro Studi di Osteopatia Tradizionale
33. AEMO- Accademia europea di medicina osteopatica
34. EICO Italia European Institute of Classical Osteopathy
35. ASFOM
36. A.I.F.R.O.M.M. - Accademia Internazionale di Formazione e Ricerca in Osteopatia e Medicina Manuale
37. INFROP - Istituto Nazionale per la Formazione e la Ricerca in Osteopatia e Posturologia
38. OSCE - Osteopathic Spine Center Education
39. E.I.O. - European Institute of Osteopathy
40. I.C.O.M. - International College of Osteopathic Medicine
41. I.E.M.O. - Istituto Europeo per la Medicina Osteopatica
42. N.S.O. - Nuova Scuola di Osteopatia di Breath of Life
43. A.I.M.O. - Accademia Italiana Medicina Osteopatica
44. Synapsy - Arti ausiliarie delle professioni sanitarie
45. S.Os.I. - Still Osteopathic Institute
46. ASOMI - Accademia di Osteopatia
47. IRMO - Istituto Romano di Medicina Osteopatica
48. NCO - Scuola di Osteopatia
49. Fulcro - Istituto Osteopatico
50. SOFI - Scuola Franco Italiana di Osteopatia
51. S.I.F.O. - Scuola Italiana Formazione Osteopatia
52. CSO – Centro Studi di Osteopatia
53. CEESO Venezia – Centre Européen d’Enseignement Supérieur de l'Ostéopathie
54. AMOS - Accademia Medicina Osteopatica Superiore
55. Scuola Franco Italiana di Osteopatia
56. Se estero, in quale Paese?
57. Francia
58. Regno Unito
59. Spagna
60. Germania
61. Svizzera
62. Austria
63. Croazia
64. Belgio
65. Olanda
66. Paesi scandinavi
67. Stati Uniti d’America
68. Australia
69. Ha frequentato un corso part-time o full-time?
70. Part-time
71. Full-time

10A) Qual'è stata la durata del corso (anni)?

1. 1
2. 2
3. 3
4. 4
5. 5
6. 6
7. E’ in possesso di una laurea?
8. Si
9. No
   - 1. Se si:
        1. Sanitaria
        2. Non sanitaria
10. Nello specifico, qual è la sua precedente formazione?
11. Fisioterapia
12. Medicina
13. Massofisioterapia
14. Scienze motorie
15. Scienze infermieristiche
16. ISEF
17. Ostetricia
18. Veterinaria
19. Psicologia
20. Biologia
21. Nessuna delle precedenti
22. Altro [campo libero]
23. Quali titoli ha acquisito? Consentita più di una risposta
24. D.O.
25. D.O. (USA)
26. Eur Ost D.O.
27. M.R.O.
28. R.O.
29. BSc Ost.
30. MSc Ost.
31. Dott.
32. Ph.D.
33. Altro
34. Frequenta corsi di aggiornamento?
35. Si
36. no
    - - 1. Se si, di media, quanti all'anno?

- 1
- 2
- 3
- 4
- 5
- 6
- 7
- 8
- 9
- 10
- 11
- 12
- 13
- 14
- 15
- 16
- 17
- 18
- 19
- 20

Legge articoli scientifici su riviste osteopatiche?

- Si
- no

Se si, con quale frequenza:

- giornaliera
- settimanale
- mensile
- trimestrale
- semestrale
- annuale

Pratica Clinica

1. Da quanti anni lavora come osteopata?
    - 1
   - 2
   - 3

- ...
- 50

1. Oltre all’osteopata, lavora anche come altro?
2. Si
3. no
   - 1. Se si, cosa fa? più di una risposta consentita
        - fisioterapista
        - medico
        - massofisioterapista
        - scienze motorie
        - infermiere
        - ISEF
        - ostetrica
        - veterinario
        - biologo
        - psicologo
        - amministratore di una scuola di osteopatia
        - direttore generale/dipartimento in una scuola di osteopatia
        - insegnate/assistente presso uno o più istituti di osteopatia
        - tutor/assistente presso uno o più istituti di osteopatia
        - supervisore tesi/ricerca presso uno o più istituti di osteopatia
        - docente di corsi di formazione post-graduate in ambito osteopatico
        - occasionalmente lavora come docente presso uno o più istituti di osteopatia
        - amministratore di altra società
        - altro:
4. Quanti giorni la settimana lavora come osteopata?
   1. 1
   2. 2
   3. 3
   4. 4
   5. 5
   6. 6
   7. 7
5. Quante ore al giorno (in media) visita pazienti come osteopata?
6. 1
7. 2
8. 3
9. 4
10. 5
11. 6
12. 7
13. 8
14. 9
15. 10
16. 11
17. 12
18. 13
19. 14
20. 15
21. 16
22. 17
23. 18
24. Lavora come:
25. Osteopata dipendente retribuito
26. Osteopata libero professionista
27. Osteopata libero professionista, non nel proprio studio
28. Dove lavora? Consentita più di una risposta
29. Proprio studio
30. Clinica privata
31. Presidio ospedaliero
32. Scuola di osteopatia
33. Università
34. Altro
35. Lavora da solo o associato con altri specialisti?
36. Solo
37. Associato
38. Se associato, da quante persone è composto lo studio associato?
39. 2
40. 3
41. 4
42. 5
43. >5
44. Se lavora in uno studio associato, quali altri professionisti sono presenti? [Più di una risposta consentita]
45. Osteopati
46. Medici di base
47. Fisioterapisti
48. Terapisti occupazionali
49. Psicologi
50. Logopedisti
51. Dietisti/dietologi
52. Ortodonzisti/odontoiatri
53. Massaggiatori
54. Medici specialisti
55. Optometristi
56. Altro:
57. In quale provincia italiana lavora? Consentita più di una risposta
58. Elenco province

In quali altre nazioni, lavora come osteopata? Risposta uguale alla 9 + altro

1. Quanto tempo dedica ad una nuova visita?
2. <30 min
3. 30-45 min
4. 46-60 min
5. >60 min
6. Quanto tempo dedica ad un controllo?
   1. <30 min
   2. 30-45 min
   3. 46-60 min
   4. >60 min
7. Appena finita una visita, pianifica immediatamente la successiva?
8. Si
9. No
10. Non sempre
11. Pianifica una serie di appuntamenti tutti insieme per lo stesso paziente?
12. Si
13. No
14. Non sempre
15. Effettua una nuova valutazione diagnostica osteopatica ad ogni visita?
16. Si
17. No
18. Non sempre
19. Quali modalità diagnostiche osteopatiche usa? Se una modalità è sconosciuta indichi “mai”

0=mai; 1=raramente; 2=a volte; 3=spesso; 4=sempre

| modalità | 0 | 1 | 2 | 3 | 4 |
| --- | --- | --- | --- | --- | --- |
| valutazione della mobilità viscerale |  |  |  |  |  |
| valutazione craniale (neuro e viscerocranio) |  |  |  |  |  |
| valutazione fasciale |  |  |  |  |  |
| ispezione |  |  |  |  |  |
| Test della funzione muscolare |  |  |  |  |  |
| test riflessi neurolinfatici |  |  |  |  |  |
| valutazione strutturale |  |  |  |  |  |
| valutazione movimento presente |  |  |  |  |  |
| Percussione e ascultazione |  |  |  |  |  |
| Range of motion (ROM) |  |  |  |  |  |
| tender points e trigger points |  |  |  |  |  |
| test ortopedici classici |  |  |  |  |  |
| test neurologici classici |  |  |  |  |  |
| altro |  |  |  |  |  |

1. Quali tecniche osteopatiche usa? se una tecnica è sconosciuta indichi “mai”

0=mai; 1=raramente; 2=a volte; 3=spesso; 4=sempre

| tecnica | 0 | 1 | 2 | 3 | 4 |
| --- | --- | --- | --- | --- | --- |
| automatic shifting e approccio fluidico |  |  |  |  |  |
| fasciale (rilascio miofasciale, chila techniques, ‘unwinding’) |  |  |  |  |  |
| fluidiche (pompaggio linfatico …) |  |  |  |  |  |
| funzionali (Sutherland, Hoover, Jones or counterstrain techniques, Balanced Ligamentous Tension techniques (BLT), ...) |  |  |  |  |  |
| mobilizzazione osteopatiche generali (General Osteopathic Treatment (GOT), Total Body Adjustment (TBA)) |  |  |  |  |  |
| Alta velocita bassa ampiezza (HVLA- techniques, impulse manipulations) |  |  |  |  |  |
| muscolari (i.e. Mitchell) |  |  |  |  |  |
| neurocraniali e viscerocraniali |  |  |  |  |  |
| tecniche dei riflessi neurocraniali e neurolinfatiche |  |  |  |  |  |
| tecniche di percussione/vibrazione |  |  |  |  |  |
| trigger points |  |  |  |  |  |
| Progressive Inhibition of Neuromuscular Structures (PINS) |  |  |  |  |  |
| tecniche sui tessuti molli e connettivali |  |  |  |  |  |
| viscerali |  |  |  |  |  |
| toggle |  |  |  |  |  |
| altro |  |  |  |  |  |

1. Fa uso di tecniche osteopatiche interne?

0=mai; 1=raramente; 2=a volte; 3=spesso; 4=sempre

| tecnica | 0 | 1 | 2 | 3 | 4 |
| --- | --- | --- | --- | --- | --- |
| intrabuccale |  |  |  |  |  |
| vaginale |  |  |  |  |  |
| rettale |  |  |  |  |  |

1. Normalmente, durante una visita, suggerisce ulteriori consigli riguardo lo stile di vita del paziente? Consentita più di una risposta

0=mai; 1=raramente; 2=a volte; 3=spesso; 4=sempre

|  | 0 | 1 | 2 | 3 | 4 |
| --- | --- | --- | --- | --- | --- |
| attività fisica |  |  |  |  |  |
| alimentari |  |  |  |  |  |
| ergonomici |  |  |  |  |  |
| stile di vita |  |  |  |  |  |

1. Preferisce visitare specifici gruppi di pazienti?
2. Si
3. no
   - 1. Se si, quali? [Consentita più di una risposta]
        1. infanti
        2. bambini
        3. donne incinte
        4. atleti
        5. anziani
        6. altro:

1. Usa metodi diagnostici e/o terapeutici aggiuntivi, per i quali si è formato, durante la pratica clinica? e.g. chinesiologia applicata, bioterapia nutrizionale, agopuntura, fitoterapia, omeopatia
2. Si
3. No
   - 1. Se si, quali [campo libero]
4. Come promuove la sua pratica clinica? [Consentita più di una risposta]
5. Sito web personale
6. Brochure
7. Collaborazione con altri specialisti
8. Pubblicità cartacea
9. pubblicità su siti internet
10. Biglietti da visita
11. Promozione attraverso l’iscrizione su registri di professionisti
12. Conferenze
13. Altro
14. Ha tariffe diverse in base a pazienti differenti?
15. Si
16. No
17. Quale è la sua tariffa in € per una prima visita? Se ha tariffe diverse, indichi la tariffa più elevata.
18. <25
19. 26-30
20. 31-40
21. 41-50
22. 51-60
23. 61-70
24. 71-80
25. 81-90
26. 91-100
27. >100
28. Quale tariffa applica per la visita successiva?
29. <25
30. 26-30
31. 31-40
32. 41-50
33. 51-60
34. 61-70
35. 71-80
36. 81-90
37. 91-100
38. >100
39. Applica sconti in caso di richiesta del paziente?
40. Si
41. No

I vostri pazienti

1. Qual è il tempo di attesa medio per una prima visita per un paziente?
2. Stesso giorno
3. Entro 1 settimana
4. 1-2 settimana
5. 2-3 settimane
6. 3-4 settimane
7. > 4 settimane
8. Quanti pazienti visita in media durante una giornata lavorativa?
9. 0-5
10. 6-10
11. 11-15
12. 16-20
13. >20
14. Quanti pazienti visita in media durante una settimana lavorativa?
15. <5
16. 6-10
17. 11-15
18. 16-20
19. 21-25
20. 26-30
21. 31-35
22. 36-40
23. 41-45
24. 46-50
25. 51-55
26. >55
27. Quante nuove visite ha in media a settimana? Per nuova visita si intende un paziente che non è stato mai trattato da Lei.
28. 0-5
29. 6-10
30. 11-15
31. 16-20
32. >20
33. Qual è la media (indicativa) del numero di trattamenti per ogni motivo di consultazione? Il paziente cronico non è incluso.
34. Campo numerico da 1 a 40
35. I suoi pazienti sono principalmente:
36. Uomini
37. Donne
38. Divisi ugualmente
39. Non so

- Tratta anche animali?

- Si

- no

- Se si, in media quante ore la settimana dedica al trattamento degli animali?

1. Come vengono a conoscenza di lei i pazienti? Più di una domanda può essere scelta.

0=mai; 1=raramente; 2=a volte; 3=spesso; 4=sempre

|  | 0 | 1 | 2 | 3 | 4 |
| --- | --- | --- | --- | --- | --- |
| Mandati da un medico |  |  |  |  |  |
| Mandati da un fisioterapista |  |  |  |  |  |
| Mandati da un collega-osteopata |  |  |  |  |  |
| Su consiglio di un altro paziente |  |  |  |  |  |
| Su consiglio di un loro conoscente |  |  |  |  |  |
| Su iniziativa del paziente stesso (via internet, pubblicità, brochure, ecc…) |  |  |  |  |  |
| Non so |  |  |  |  |  |
| Altro |  |  |  |  |  |

1. Negli ultimi 6 mesi ha trattato pazienti di quale età: [più di una risposta può essere scelta].
2. Meno di 6 mesi
3. 6 mesi - 2 anni
4. 2 - 10 anni
5. 11 - 20 anni
6. 21 - 40 anni
7. 41 - 64 anni
8. 65 anni e oltre
9. Non so
10. La maggioranza dei pazienti ha: [3 risposte al massimo].
11. Meno di 6 mesi
12. 6 mesi a 2 anni
13. 2 a 10 anni
14. 11 a 20 anni
15. 21 a 40 anni
16. 41 a 64 anni
17. 65 anni e oltre
18. Non so
19. Per quali problematiche (per regione corporea) viene maggiormente contattato dai pazienti?

0: mai 1: raramente 2: regolarmente 3: spesso 4: sempre

| testa | 0 | 1 | 2 | 3 | 4 |
| --- | --- | --- | --- | --- | --- |
| colonna cervicale |  |  |  |  |  |
| cingolo scapolare |  |  |  |  |  |
| gomito |  |  |  |  |  |
| polso/mano |  |  |  |  |  |
| colonna toracica |  |  |  |  |  |
| torace |  |  |  |  |  |
| addome |  |  |  |  |  |
| colonna lombare |  |  |  |  |  |
| bacino |  |  |  |  |  |
| anca |  |  |  |  |  |
| ginocchio |  |  |  |  |  |
| caviglia/piede |  |  |  |  |  |
| altro |  |  |  |  |  |

1. Per quali disturbi nei seguenti ambiti clinici è stato maggiormente contattato dai pazienti nell’ultimo anno?

0=mai; 1=raramente; 2=a volte; 3=spesso; 4=sempre

| Distorsioni cavilgia | 0 | 1 | 2 | 3 | 4 |
| --- | --- | --- | --- | --- | --- |
| cervicobrachialgia |  |  |  |  |  |
| Problematiche durante/post gravidanza e nascita |  |  |  |  |  |
| Problematiche craniomandibolari |  |  |  |  |  |
| Bambini irrequieti |  |  |  |  |  |
| Problemi dermatologici (es. eczema, psoriasi, etc..) |  |  |  |  |  |
| Disturbi digestivi |  |  |  |  |  |
| Instabilità-vertigini |  |  |  |  |  |
| otiti |  |  |  |  |  |
| fibromialgia |  |  |  |  |  |
| Problematiche di spalla |  |  |  |  |  |
| Reflusso gastro-esofageo |  |  |  |  |  |
| Cefalea-emicrania |  |  |  |  |  |
| incontinenza |  |  |  |  |  |
| Sindrome dell'intestino irritabile |  |  |  |  |  |
| Lombalgia |  |  |  |  |  |
| Problematiche meniscali |  |  |  |  |  |
| Dolori mestruali |  |  |  |  |  |
| Dolori cervicali |  |  |  |  |  |
| Problematiche post-chirurgiche |  |  |  |  |  |
| Disturbi psicologici |  |  |  |  |  |
| sciatalgia |  |  |  |  |  |
| Disturbi del sonno |  |  |  |  |  |
| Disturbi legati allo stress |  |  |  |  |  |
| Stanchezza |  |  |  |  |  |
| altro |  |  |  |  |  |

Commenti

1. Avete altri commenti e/o note ulteriori riguardo questo questionario? [Campo libero]
2. Qual è la vostra idea su uno sviluppo perfetto della professione di osteopata? [Campo libero]
